# Senescent macrophages in the human adipose tissue as a source of inflammaging

**DOI:** 10.1007/s11357-022-00536-0

**Published:** 2022-03-05

**Authors:** Giulia Matacchione, Jessica Perugini, Eleonora Di Mercurio, Jacopo Sabbatinelli, Francesco Prattichizzo, Martina Senzacqua, Gianluca Storci, Christian Dani, Giovanni Lezoche, Mario Guerrieri, Antonio Giordano, Massimiliano Bonafè, Fabiola Olivieri

**Affiliations:** 1 Department of Clinical and Molecular Sciences, DISCLIMO, Università Politecnica delle Marche, Via Tronto 10/A, Ancona, Italy; 2 Department of Experimental and Clinical Medicine, Center of Obesity, Università Politecnica delle Marche, Ancona, Italy; 3 IRCCS MultiMedica, Milano, Italy; 4 IRCCS Azienda Ospedaliero-Universitaria di Bologna, Bologna, Italy; 5 Inserm, iBV, Faculté de Médecine, Université Côte d’Azur, CNRS, Nice Cedex, France; 6 Department of General Surgery, Università Politecnica delle Marche, Ancona, Italy; 7 Department of Experimental, Diagnostic and Specialty Medicine, Università di Bologna, Bologna, Italy; 8 Center of Clinical Pathology and Innovative Therapy, IRCCS INRCA, Ancona, Italy

**Keywords:** Senescent cell, Macrophage, Obesity, Insulin resistance, Inflammaging, Adipose tissue

## Abstract

Obesity is a major risk factor for type 2 diabetes and a trigger of chronic and systemic inflammation. Recent evidence suggests that an increased burden of senescent cells (SCs) in the adipose tissue of obese/diabetic animal models might underlie such pro-inflammatory phenotype. However, the role of macrophages as candidate SCs, their phenotype, the distribution of SCs among fat depots, and clinical relevance are debated. The senescence marker β-galactosidase and the macrophage marker CD68 were scored in visceral (vWAT) and subcutaneous (scWAT) adipose tissue from obese patients (*n*=17) undergoing bariatric surgery and control patients (*n*=4) subjected to cholecystectomy. A correlation was made between the number of SCs and BMI, serum insulin, and the insulin resistance (IR) index HOMA. The monocyte cell line (THP-1) was cultured in vitro in high glucose milieu (60 mM D-glucose) and subsequently co-cultured with human adipocytes (hMADS) to investigate the reciprocal inflammatory activation. In obese patients, a significantly higher number of SCs was observed in vWAT compared to scWAT; about 70% of these cells expressed the macrophage marker CD68; and the number of SCs in vWAT, but not in scWAT, positively correlated with BMI, HOMA-IR, and insulin. THP-1 cultured in vitro in high glucose milieu acquired a senescent-like phenotype (HgSMs), characterized by a polarization toward a mixed M1/M2-like secretory phenotype. Co-culturing HgSMs with hMADS elicited pro-inflammatory cytokine expression in both cell types, and defective insulin signaling in hMADS. In morbid obesity, expansion of visceral adipose depots involves an increased burden of macrophages with senescent-like phenotype that may promote a pro-inflammatory profile and impair insulin signaling in adipocytes, supporting a framework where senescent macrophages fuel obesity-induced systemic inflammation and possibly contribute to the development of IR.

**Supplementary Information:**

The online version contains supplementary material available at 10.1007/s11357-022-00536-0.

## Introduction

Inflammaging is a portmanteau word that was minted to depict the chronic, sterile, low-grade inflammatory status that characterizes the human aging process [1, 2]. A state of systemic hyper-inflammation has been also reported in a large number of age-related dysfunctions and diseases (ARDs), such as in obese subjects and in people affected by type 2 diabetes mellitus, T2DM [3, 4]. In turn, overnutrition/obesity and chronological aging can synergize in accelerating the rate of inflammaging, thus suggesting that inflammatory mechanisms at the basis of insulin resistance (IR)/T2DM, obesity, and aging are very difficult to disentangle [5, 6]. In the recent years, age-related accrual of senescent cell (SC) burden in vivo gained the spotlight, as it was demonstrated that their selective ablation in mice is capable to delay aging features in tissue and organs [7]. In vitro, the loss of replicative capacity in SCs is accompanied by the onset of a pro-inflammatory senescence-associated secretory phenotype (SASP) [8]. In vivo, SASP is indicted to contribute to inflammaging and to play a crucial role in the onset and prognosis of diabetic complications [9–11].

Among SASP factors, a small number of miRNA, named inflamma-miRs and comprising miR-21, and miR-146a, are involved in regulating both cellular senescence and organismal inflammaging [12, 13]. During aging, inflamma-miR expression was found to be upregulated to restrain the tissue damage induced by the low-level chronic inflammation. The abnormal level of miR-21 and miR-146a has been observed both in normal aging and in the major ARDs [14, 15]. In both mice model and observational studies in humans, miR-21 and miR-146a expression levels were consistently associated with both obesity and T2DM [16–19].

White adipose tissue (WAT) cells are highly susceptible to undergo senescence not only with aging but also with obesity and T2DM, independently of chronological age [20]. WAT senescence is associated with dysfunctional expansion (hypertrophy) of adipocytes, IR, and dyslipidemia. Recently, SCs have been found to accumulate in the adipose tissue of obese and diabetic human and mice [21, 22].

In the adipose tissue, features of senescence, such as a higher level of senescence-associated β-galactosidase (SA-β-Gal) activity, and p53 and p21 protein levels have been documented [23, 24]. Of note, the increase in the SC burden observed in diabetic mice was associated with impaired glucose tolerance, accrual of macrophages in the adipose tissue, and elevated production of pro-inflammatory mediators, while SC removal or attenuation of their phenotype improved metabolic homeostasis [11, 25]. However, few studies have explored the burden of SCs in human fat samples and their relevance in relation to IR [9]. In addition, the cell type responsible for the SC burden observed in fat tissue is still debated, being pre-adipocytes, fully differentiated adipocytes, endothelial cells, macrophages, or other infiltrating immune cells possible candidates [26]. To address these issues, we aimed at identifying and quantifying the burden of senescent macrophages in visceral and subcutaneous adipose tissue samples obtained from obese and control patients, exploring their correlation with IR. Then, we exposed the human monocytic cell line (THP-1) to high glucose, an over-nutrition mimicking stimulus [10], to characterize the resulting phenotype and to set up a co-culture with human multipotent adipose-derived stem cells (hMADS), in order to evaluate the reciprocal influence on inflammation and insulin sensitivity.

## Materials and methods

### Patients

For histological analysis, adipose tissue (AT) biopsies were obtained from 17 severely obese patients (BMI≥ 35 kg/m^2^, i.e., obesity class II) and 4 control subjects (BMI ≤30 kg/m^2^) undergoing respectively bariatric and non-bariatric (i.e., cholecystectomy) surgery at the General Hospital Azienda Ospedaliera Universitaria (AOU Ospedali Riuniti; Ancona, Italy). Samples from visceral (vWAT, omental depot) and subcutaneous (scWAT, abdominal region) adipose tissue were collected. The study protocol was approved by the Ethics Committee of Ospedali Riuniti (Ancona, Italy). Obesity was diagnosed and classified according to the BMI criteria. Subjects on anti-inflammatory drugs were excluded from the study. The information collected included anthropometric data and medical history (Table 1).

**Table 1:** Comparison of biochemical and anthropometric characteristics among normal weight and obese subjects

Groups	Normal weight	Obese
Sex, M/F, N/N	0/4	6/11
Age, year	76.3 ± 4.6	47.6 ± 2.2
Body mass index, kg/m^2^	27.8±2.3	45.58±0.91
Total cholesterol, mg/ml	180±5.2	180.2 ± 8.4
Triglycerides, mg/dl	98.4±8.3	121.9 ± 9.9
HbA1c, %	5.7±0.4	6.56 ± 0.7
Fasting glucose, mg/ml	93.7 ± 6.3	100.33 ± 4.4
Fasting insulin, mcUI/ml	5.4±1.7	35.5± 2.7
HOMA index	1.25±0.9	8.2±5.1

*N* is 4 for normal-weight group and 17 for obese group. Variables are expressed as mean ± SEM

### SA-β-galactosidase staining for fresh tissue and morphometry

AT biopsies (3–5-mm thickness) were fixed for 1 h with a fixative solution provided by the Senescence detection kit (Biovision Inc., Milpitas, CA, USA). After washing with PBS, samples were stained with the staining solution (X-Gal substrate: 20 mg/ml), provided by the Senescence detection kit (Biovision), and incubated at 37°C (without CO_2_) for 20–22 h. After, AT samples were first fixed in 4% paraformaldehyde overnight at 4 °C and then embedded in paraffin blocks.

From each adipose specimen, serial paraffin sections (4 μm thick) were obtained and counterstained with Fast Red (Sigma-Aldrich, Milano, Italy). Tissue sections were examined with a Nikon Eclipse E800 light microscope and digital images were captured with a Nikon camera (DXM 1200). Senescence-associated (SA)-β-galactosidase (Gal) positive (+) cells and adipocytes were counted by optical microscopy. The number of senescent cells was expressed as percentage in relation to adipocytes by counting the number of SA-β-Gal + cells and the number of adipocytes in at least 20 fields. The analysis excluded cells within and around blood vessels.

### *CD68 *and* p16*^*INK4a*^immunostaining

For immunohistochemistry, fixed AT samples were embedded with paraffin. Paraffin sections, 4 μm thick, were reacted with 3% hydrogen peroxide in PBS for 10 min, and antigen-retrieved using citrate buffer (ph 6.0) in a steamer for 45 min. After blocking with 3% horse normal serum, sections were incubated overnight at 4°C with anti-CD68 (Dako, Glostrup, Denmark) primary antibody (1:200) and p16^INK4a^ (ThermoFisher Scientific, Massachusetts, USA) primary antibody (1:800). Bound primary antibody was detected using a biotinylated anti-mouse IgG secondary antibody, ABC reagent (Vector Laboratories, Burlingame, USA), and DAB Substrate kit (Sigma-Aldrich) according to the manufacturer’s instructions. Finally, sections were counterstained with Fast Red and mounted. CD68 + cells were counted among SA-β-Gal + cells in the entire tissue section. The analysis excluded cells within and around blood vessels.

### THP-1 culture, macrophage polarization, and high-glucose concentration exposure treatment

Human monocytic THP-1 cells were purchased from ATCC (Rockville, MD, USA) and maintained in RPMI-1640 medium supplemented with 2-mercaptoethanol to a final concentration of 0.05 mM and with 10% heat-inactivated fetal bovine serum (FBS), 1× penicillin/streptomycin (100×), and 1× l-glutamine (100×) (all from Euroclone, Milano, Italy).

To induce polarization of THP-1 cells into the M0 phenotype macrophages, cells were treated with phorbol 12-myristate 13-acetate (PMA) (100 ng/ml) for 24 h. Afterwards, LPS (15 ng/ml) in RPMI medium with 10% FBS was added for 48 h to polarize M0 macrophages into the M1 phenotype (pro-inflammatory), whereas the M2 phenotype (anti-inflammatory) was induced with IL-4 (25 ng/ml) and IL-13 (25 ng/ml) in RPMI medium with 10% FBS for 48 h. THP-1 cells were maintained in high-glucose medium (60 mM) for 1 week and then treated with 100 ng/ml PMA for 24 h, to mimic hyperglycemia and to obtain macrophages (HgSMs). In order to examine THP-1 proliferation after 1 week treatment with 60 mM d-glucose, cells were plated in 12-well plates at a density of 200,000 cells/ml, treated without or with 60 mM of d-glucose (NG and HG respectively) and then counted after 1 week treatment. The experiment was performed in triplicate.

### Cell viability assay

MTT (3-(4,5-dimethylthiazol-2-yl)-2,5-diphenyltetrazolium bromide) assay was used to test cell viability and proliferation of THP-1 cells treated with 60 mM d-glucose. Cells were grown in 96-well plates at a density of 10000 cells/well and treated with or without 60 mM of d-glucose. After 1 week, MTT (1 mg/ml) solution was added and incubated for 4 h; the insoluble formazan salt product was solubilized by adding 200 μl of dimethyl sulfoxide (DMSO) and its amount was determined by measuring the optical density at 540 nm using a microplate reader (MPT Reader, Invitrogen, Milano, Italy).

Cell viability was calculated according to the equation (*T*/*C*) × 100%, where *T* and *C* represent respectively the mean optical density of the treated group and the control group.

### hMADS cell culture and adipocyte differentiation

hMADS cells were cultured and differentiated as previously described [27]*.* Briefly, hMADS cells were grown in low-glucose (1 g/l) proliferation medium (DMEM) supplemented with 10% fetal bovine serum and 2.5 ng/ml hFGF-2. For differentiation, hMADS cells were used between the 16th and the 19th passage. To induce adipose differentiation, they were seeded in proliferation medium on 6-well cell culture plates at a density of 4500 cells/cm^2^. When they reached confluence, hFGF-2 was not replaced. The next day (day 0), cells were incubated in adipogenic medium and cell lipid content was assessed by Oil Red O staining as previously described [27].

### hMADS adipocytes and THP-1 co-culture system

hMADS cells were grown to confluency in 6-well cell culture plates and differentiated to mature adipocytes as described above. Adipocytes were then co-cultured in a transwell system with THP-1 cells differentiated into M0, M1, M2, and HgSMs as described above. THP-1 cell activation and polarization were performed in a 0.4-μm cell inserts and cultured with hMADS adipocytes in the lower chamber. For controls, cells were cultured individually. Co-culture experiments were carried on for 48 h.

### SA-β-galactosidase staining for cell culture

SA-β-Gal activity was detected by using Senescence Detection Kit (Biovision). Briefly, M0 and HgSM cultured in 12-well plates were fixed for 15 min at room temperature and then washed twice in PBS. Cells were incubated overnight at 37°C with Staining Solution Mix (containing X-gal).

### Cytokine releasing measurement

Condition media were collected at the end of each incubation, centrifuged, and stored at −80°C until use in the assays. IL-6 and IL-1β concentrations (pg/ml) were measured using commercially available, high-sensitivity ELISA kits (Cayman Chemical and Finetest) according to the manufacturer’s instructions.

### mRNA and mature miRNA quantitative RT-PCR

Total RNA was isolated using the Norgen Biotek Kit (Thorold, ON, Canada), according to the manufacturer’s instructions. For adipose tissue, a first step with Trizol (ThermoFisher Scientific, Massachusetts, USA) and chloroform (Sigma-Aldrich) was performed to remove lipid content. RNA was stored at −80 °C until use.

RNA amount was determined by spectrophotometric quantification with Nanodrop ONE (NanoDrop Technologies, Wilmington, DE, USA). Total RNA was reverse-transcribed using PrimeScript RT reagent Kit (Takara bio, Japan) according to the manufacturer’s instructions. qRT-PCR was performed in a Rotor-Gene Q (Qiagen Hilden, Germany) using TB Green Premix Ex Taq (Takara bio, Japan). All primers (Table 2) were from Merck Millipore (Darmstadt, Germany). Each single sample of the biological triplicate was loaded in a technical duplicate into the real-time plate. GAPDH and β-actin were used as endogenous controls. mRNA expression was assessed using the 2^−DDCt^ method.

**Table 2:** Primers and TaqMan miRNA assays

Target gene	Forward	Reverse
ADIPOQ	GAGATGGACGGACGGAGTCCTTTAGG	CTGGTCATGTTTGTGAAGCTCCC
β-actin	TGAGAGGGAAATCGTGCGTG	TGCTTGCTGATCCACATCTGC
CD206	TATGGAATAAAGACCCGCTGAC	TGCTCATGTATCTCTGTGATGCT
GAPDH	GGCACAGTCAAGGCTGAGAATG	ATGGTGGTGAAGACGCCAGTA
GLUT4	CATTCCTTGGTTCATCGTG	ATAGCCTCCGCAACATAC
IL-1β	AGATGATAAGCCCACTCTACAG	ACATTCAGCACAGGACTCTC
IL-6	TGCAATAACCACCCCTGACC	GTGCCCATGCTACATTTGCC
IL-8	GGACAAGAGCCAGGAAGAAA	CCTACAACAGACCCACACAATA
IL-10	AGGCATTCTTCACCTGCTCC	AAGACCCAGACATCAAGGCG
MCP1	GGCTGAGACTAACCCAGAAAAG	GGGTAGAAACTGTGGTTCAAGAG
NF-kB	ACAGCTGGATGTGTGACTGG	TCCTCCGAAGCTGGACAAAC
p21	CCATCCCTCCCCAGTTCATT	AAGACAACTACTCCCAGCCC
SIRT1	TGTTTCCTGTGGGATACCTGA	TGAAGAATGGTCTTGGGTCTTT
TNF-α	AAGCCTGTAGCCCACGTGTA	GGCACCACTAGTTGGTGGTCTTTG
TGF-b	CCCAGCATCTGCAAAGCT	GTCAATGTACAGCTGCCGCA
IL-1α	CTTTCCCTGCCTGACCTTATT	GAATGAAGCTACTGCCCTACTC
PPAR-g	TGTGGGGATAAAGCATCAGGC	CCGGCAGTTAAGATCACACCTAT
CD163	ACTGCAAGAACTGGCAATGG	CCATGCTTCACTTCAACAGG
	**TaqMan assay ID**	
miR-21	ID00397	
miR-146a	ID000468	
RNU44	ID001094	

The expression of miR-146a and miR-21 (Table 2) was quantified by RT-qPCR using TaqMan miRNA assays (all from ThermoFisher Scientific) according to the manufacturer’s protocol. Data were analyzed with Rotor Gene Q (Qiagen, Hilden, Germany) with the automatic comparative threshold (Ct) setting for adapting baseline. qRT-PCR data were normalized to RNU44 (Table 2). The 2^−DDCT^ method was used to determine miRNA expression.

### DNA isolation and telomere length measurement

DNA was isolated using E.Z.N.A. Tissue DNA Kit (Omega bio-tek), according to the manufacturer’s instructions. DNA was stored at −80 °C until use. The abundance of telomere signal per genome measured by qPCR represents the average telomere length in a given DNA sample.

The amount of input genomic DNA is quantified by measuring the qPCR product of a single copy gene (S) that is used to normalize the signal from the telomere (T) reaction. The single-copy gene is the 36B4, which encodes acidic ribosomal phosphoprotein PO. The resulting T/S ratio represents the average telomere length per genome.

### Protein extraction and immunoblotting

Cell lysates were obtained by using RIPA buffer (150 mM NaCl, 10 mM Tris, pH 7.2, 0.1% SDS, 1.0% Triton X-100, 5 mM EDTA, pH 8.0) containing a protease inhibitor cocktail (Roche Applied Science, Indianapolis, IN, USA). Protein concentration was determined using Bradford Reagent (Sigma-Aldrich). Total protein extracts (20 μg for hMADS and 15 μg for THP-1) were separated by SDS-PAGE and transferred to nitrocellulose membranes (Bio-Rad, Hercules, CA, USA). Membranes were then blocked for 1 h at room temperature (RT) in TBS-Tween-20 containing 5% non-fat-dried milk and subsequently incubated overnight with the primary antibody (Table 3). Finally, they were incubated with a secondary antibody conjugated to horseradish peroxidase (Vector) for 1 h at RT and bands were visualized with the Chemidoc Imaging System (Bio-Rad) using ECL Plus chemiluminescence substrate (GE Healthcare, Pittsburgh, PA, USA). Densitometric analysis was performed with ImageJ software. When appropriate, membranes were stripped by incubation at room temperature for 7 min with a mild stripping buffer (15 g of glycine, 1 g of SDS, 10 ml of Tween 20 and bring volume up to 1 l with ultrapure water). They were then washed, blocked, and re-probed for total protein content.

**Table 3:** Primary antibodies

Antibodies	Host*	Dilution	Source
AKT	R	1:1000	Cell Signaling Technology/9272
α-tubulin	R	1:1000	Cell Signaling Technology/ 2144
β-actin	M	1:200	Santa Cruz Biotechnology/sc-47778
IKB-α	R	1:1000	Cell Signaling Technology/9242
IL-1β	R	1:1000	Cell Signaling Technology/12242
IRS1	M	1:200	Santa Cruz Biotechnology/sc-8038
JNK	M	1:200	Santa Cruz Biotechnology/sc-7345
p21	R	1:1000	Cell Signaling Technology/2947
pAKT (Ser473)	R	1:2000	Cell Signaling Technology/4060
pJNK	M	1:100	Santa Cruz Biotechnology/sc-6254
SIRT1	R	1:1000	Cell Signaling Technology/2496

^*^M mouse, *R* rabbit

### Statistical analysis

Summarized data are shown as mean ± SD or as frequency (%). Independent sample *T* test was used for the analysis of real-time and densitometric data. Paired *T*-test was used to compare vWAT and scWAT. Partial correlation, adjusted for age and gender, was used to test for correlations in the obese group. Correlations between parameters were calculated using Spearman’s rho. Chi-square test was used as appropriate.

Data analysis was performed using IBM SPSS Statistics for Windows, version 25 (IBM Corp, Armonk, NY, USA). Statistical significance was defined as a two-tailed *p*-value < 0.05.

## Results

### Accumulation of SA-β-Gal + macrophages in vWAT from obese patients

**Figure 1:**
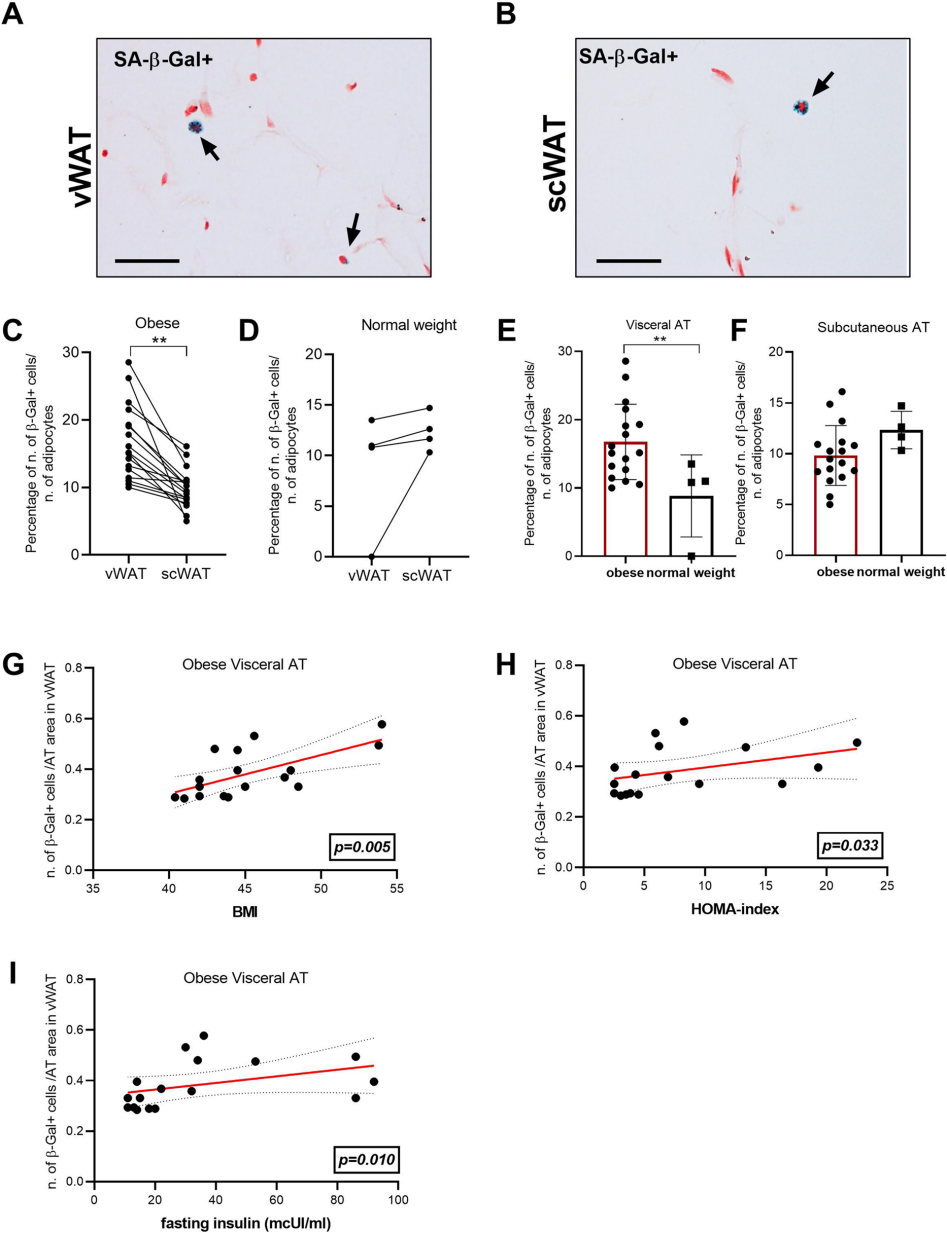
SA-β-Gal + cells in adipose tissue. Histological analysis of visceral (vWAT) (**A**) and subcutaneous adipose tissue (scWAT) (**B**) of obese subjects (*n*=17). Percentage of SA-β-Gal + cells number in vWAT and scWAT in obese subjects (**C**) and in normal weight subjects (*n*=4) (**D**). Percentage of SA-β-Gal + cells number in vWAT between obese and normal weight subjects (**E**). Percentage of SA-β-Gal + cells number in scWAT between obese and normal weight subjects (**F**). Spearman correlation between number of SA-β-Gal + cells and **G** BMI (*r*=0.671; *p*=0.003), **H** HOMA index (*r*=0.519; *p*=0.033), and **I** insulin (*r*=0.607; *p*=0.010) in vWAT. Bar= 100mm. *p≤0.05; **p≤0.01; ***p≤0.001

SCs can be identified by elevated lysosomal SA-β-Gal activity. Here, SA-β-Gal positive (+) cells were scored in visceral (vWAT, Fig. 1A) and subcutaneous (scWAT, Fig. 1B) adipose tissues of obese individuals underwent bariatric surgery (*n*=17). A significantly higher number of SA-β-Gal + cells were observed in vWAT compared to scWAT from the same obese subject (Fig. 1C). Conversely, when SA-β-Gal + cells were evaluated in a normal weight control group (*n*=4), we did not detect significant difference in the number of SA-β-Gal + cells in vWAT compared to scWAT (Fig. 1D). Notably, the comparison between obese patients and normal weight controls revealed a significantly greater amount of SA-β-Gal + cell in vWAT of obese individuals compared to controls (Fig. 1E), whereas no significant difference was found in scWAT of obese individuals compared to controls (Fig. 1F).

In obese patients, SA-β-Gal + cell number was positively correlated with BMI in vWAT but not in scWAT samples (Fig. 1G and Suppl. Fig. 1(i)). Importantly, in vWAT, SA-β-Gal + cell number was positively correlated also with the most clinically relevant markers of insulin resistance, such as HOMA index and insulin levels (Fig. 1H, I) but not in scWAT samples (Suppl. Fig. 1 (ii) and (iii)).

**Figure 2:**
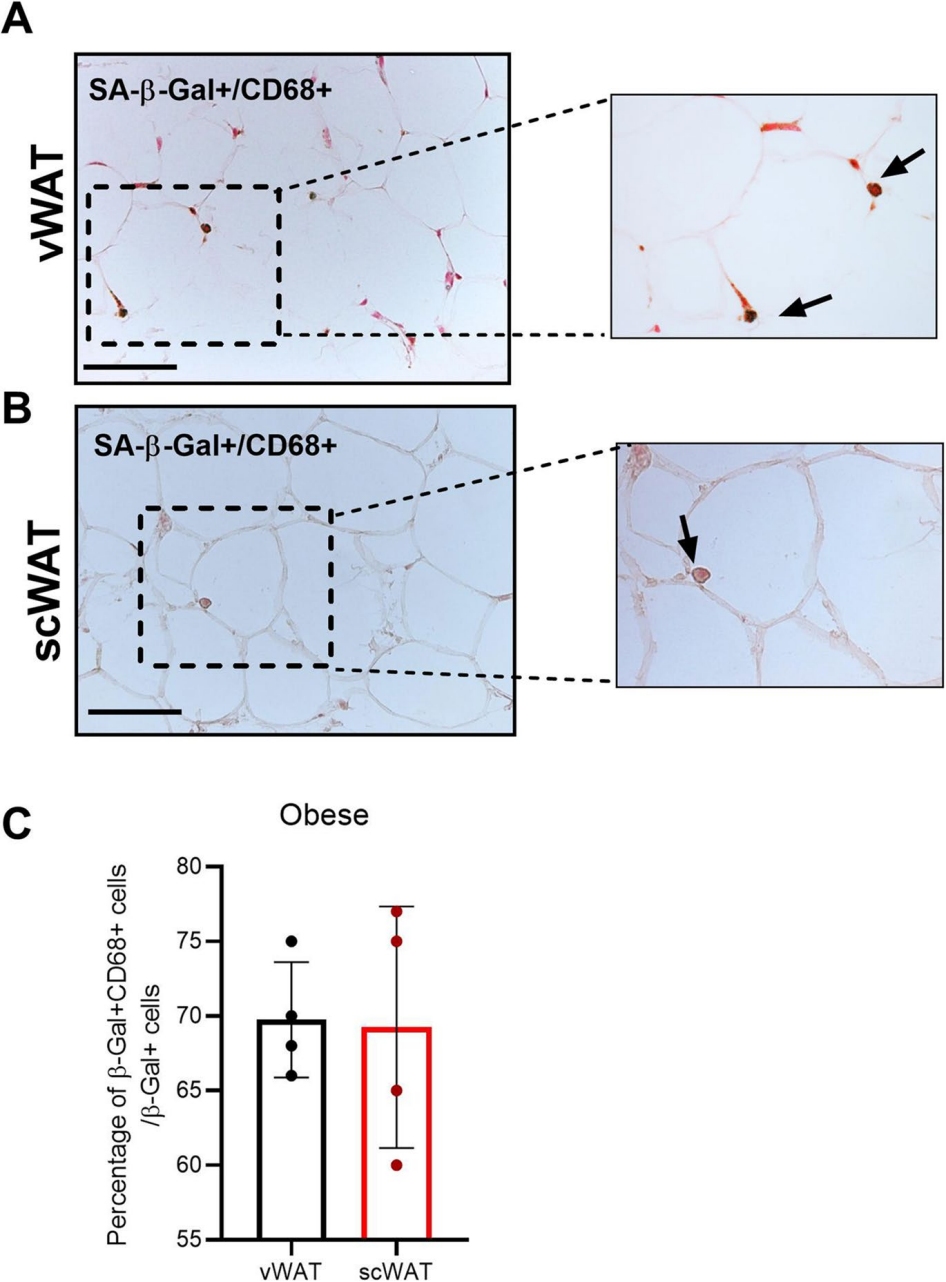
Senescent macrophages in obese adipose tissue. Immunohistochemical analysis of SA-β-Gal+/CD68+ cells in vWAT (**A**) and scWAT (**B**) of obese individuals. **C** Percentage of the double positive (SA-β-Gal+/CD68 +) cells in vWAT and scWAT of obese subjects compared to the total amount of SA-β-Gal+ cells. Bar= 100mm. *p≤0.05; **p≤0.01; ***p≤0.001

The correlations between SC number in vWAT of obese subjects and some parameters relevant in the management of diabetic patients prompted us to unravel the phenotype of senescent cells. vWAT and scWAT tissues were assessed by immunohistochemical analysis with the pan-macrophage marker CD68: the majority of SCs was positive for CD68 (Fig. 2A, B). In particular, the percentage of the double positive (SA-β-Gal/CD68 +) cells on the total amount of SA-β-Gal + cells was 69.75% in vWAT and 69.25% in scWAT of obese individuals (Fig. 2C). Moreover, an additional marker of senescence, p16^INK4a^, was performed in vWAT and scWAT of obese subjects by immunohistochemical analysis. A consistent number of β-Gal + cells resulted immunoreactive for the marker p16^INK4a^. In Supplementary Figure 2 is shown a crown-like structure (CLS) characterized by aggregates of macrophages surrounded dead adipocytes [28].

Overall, these results suggest that the accumulation of senescent macrophages in visceral adipose tissue is related to the presence of insulin resistance in obese individuals.

### THP-1 cells cultured in high glucose medium show senescent features and mixed M1/M2 phenotype

**Figure 3:**
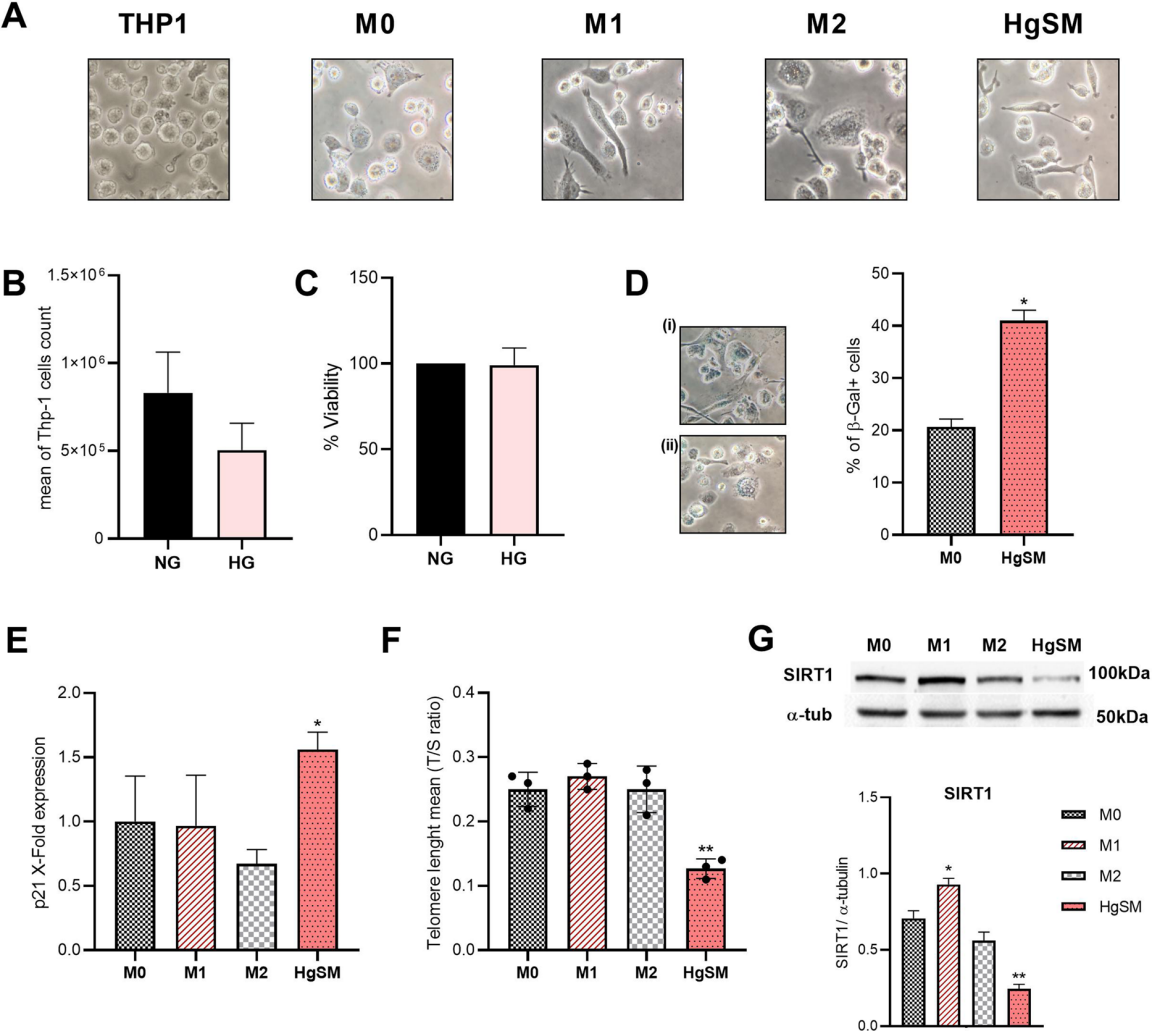
Analysis of PMA-induced macrophage phenotypes and senescent features in HgSMs. **A** Representative pictures from THP-1 cells and M0, M1, M2, and HgSM macrophage polarization observed by light microscopy (40×). **B** THP-1 proliferative activity in hyperglycemia compared to normoglycemia. **C** THP-1 viability in hyperglycemia compared to normoglycemia performed by MTT assay. **D** SA-β-Gal activity of HgSMs (i) compared to M0 (ii). **E** p21 qRT-PCR analysis in M0, M1, M2, and HgSMs. **F** Measurements of telomere length (T/S ratio) in M0, M1, M2, and HgSM macrophages. **G** Representative immunoblot and quantification of SIRT1 expression in M0, M1, M2, and HgSM macrophages. Data are expressed as mean ± SD, *p≤0.05; **p≤0.01; ***p≤0.001

To model senescent macrophages in vitro, we took advantage of the THP-1-derived macrophage model. THP-1 cells were differentiated into naïve (M0) macrophages by PMA to obtain the control condition, then polarized into M1 (by LPS) and M2 (by IL-4 and IL-13) macrophages. To mimic the exposure to hyperglycemia, THP-1 cells were cultured in the presence of high glucose concentration (60 mM) for 1 week and then induced with PMA to macrophages, henceforth named high-glucose senescent macrophages (HgSM). Morphological features of the different phenotypes were observed by optical microscopy (Fig. 3A). First, we observed no significant difference in proliferative activity and viability of THP-1 cells cultured in high-glucose medium compared to normoglycemic condition (Fig. 3B, C).

Thus, independently from proliferation, we found several senescence biomarkers in HgSMs. Namely, SA-β-Gal activity (Fig. 3D) and CDK p21 expression (Fig. 3E) were significantly higher in HgSMs than in M0 macrophages. Moreover, telomere length (Fig. 3F) and SIRT1 expression (Fig. 3G) were reduced in HgSMs compared to M0 macrophages.

**Figure 4:**
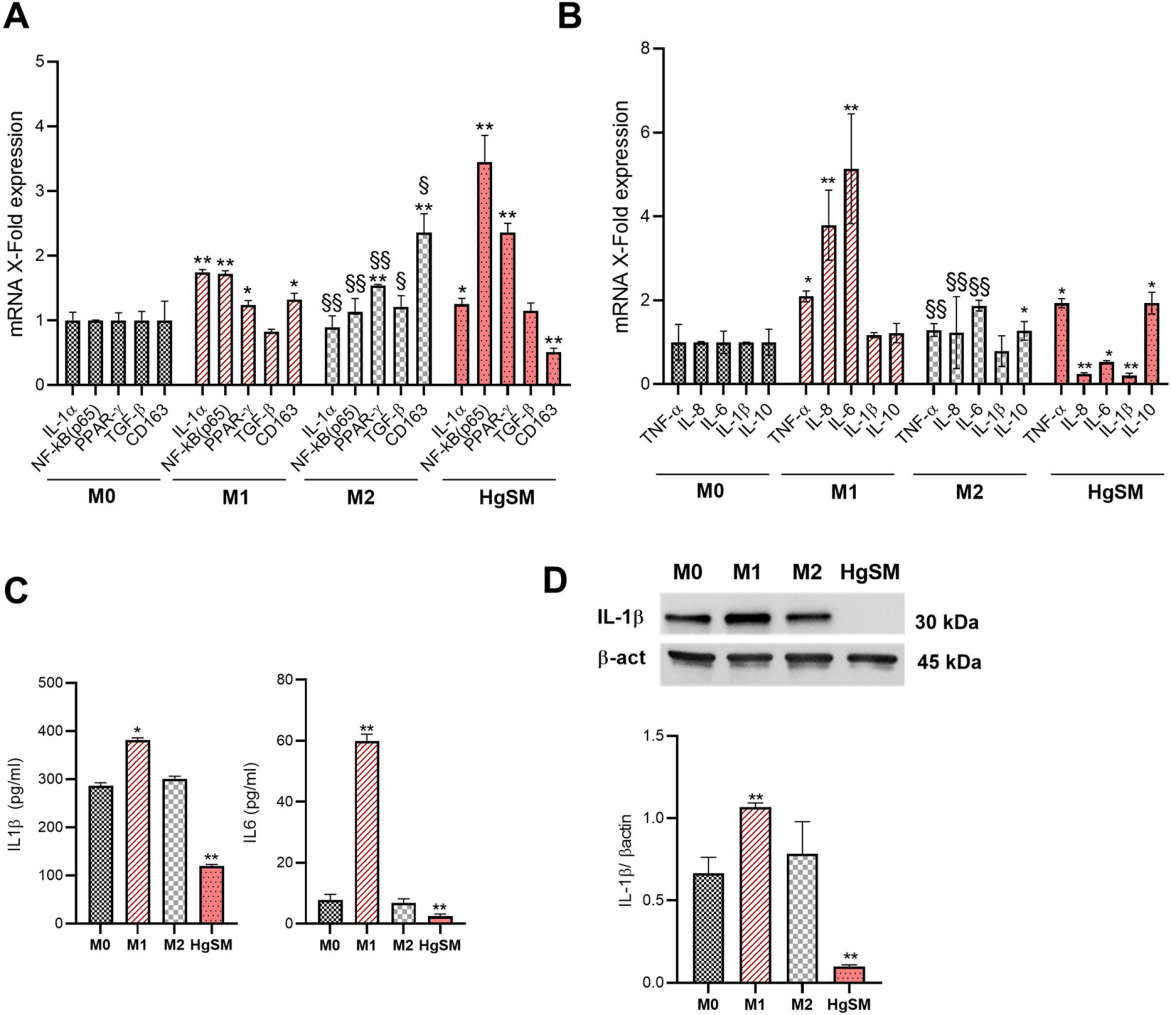
Phenotypic characterization of HgSM macrophages. **A** qRT-PCR analysis of NF-kB, Il-1a, TGF-b, PPAR-g, and CD163 expression in macrophages. **B** qRT-PCR analysis of TNF-α, IL-8, IL-6, IL-1β, and IL-10 expression in macrophages. **C** IL-1β and IL-6 concentration (pg/ml) measured by ELISA in the cell culture supernatants. **D** Representative immunoblot and quantification of IL-1β expression in M0, M1, M2, and HgSM macrophages. Data are expressed as mean ± SD, *p≤0.05; **p≤0.01; ***p≤0.001; ^§^*p*≤0.05; ^§§^*p*≤0.01; ^§§§^*p*≤0.001. *vs M0, ^§^vs M1

A number of M1 and M2 markers were analyzed in THP-1-derived macrophage, showing the appropriate differentiation of M1 and M2 macrophages (upregulation of IL-1a and NF-kB (p65), TNF-a, IL-8 and IL-6 for M1 and PPAR-g, TGF-b and CD163 for M2) (Fig. 4A, B).

Notably, HgSMs also showed a peculiar pattern of M1/M2 marker expression, dissimilar to M1- and M2-differentiated THP-1 cells, compared to controls (Fig. 4A, B). Indeed, HgSM cells expressed the highest level of NF-kB and PPAR-g as well as the lowest level of CD163 compared to M0, M1, and M2 macrophages (Fig 4A). Moreover, HgSMs conveyed a concomitant increase in pro-inflammatory TNF-α and anti-inflammatory IL-10 mRNA cytokine, coupled with a reduced expression of IL-8, IL-6, and IL-1β (Fig. 4B). Data on the low expression of IL-1β and IL-6 in HgSMs were confirmed at protein level by ELISA for both cytokines (Fig. 4C) and by Western blot analysis for IL-1β (Fig. 4D).

Collectively, our data show that high glucose triggers a senescent phenotype with M1/M2 intermediate features in THP-1- derived macrophages.

### Interplay between HgSMs macrophages and hMADS adipocytes fosters inflammation

**Figure 5:**
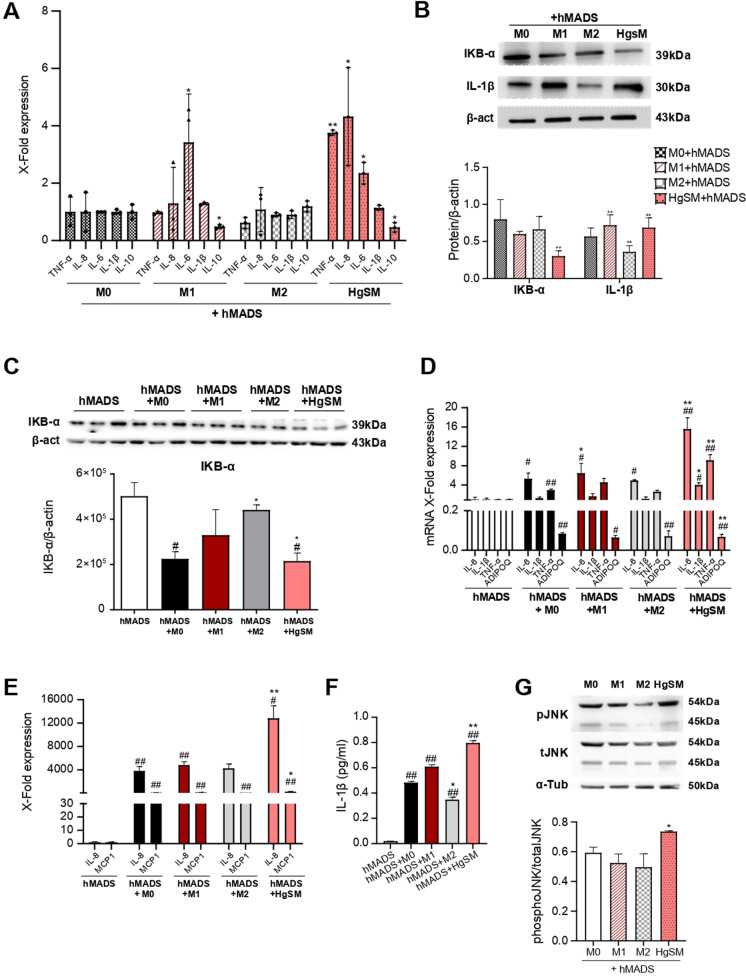
Phenotypic characterization of HgSM macrophage and hMADS adipocyte co-culture. **A** qRT-PCR analysis of TNF-α, IL-8, IL-6, IL-1β, and IL-10 expression in macrophages co-cultured with hMADS adipocytes.** B** Representative immunoblot and quantification of IKB-α and IL-1β in macrophages co-cultured with hMADS adipocytes.** C** Representative immunoblot and quantification of IKB-α in hMADS adipocytes co-cultured with macrophages. qRT-PCR analysis of IL-6, IL-1β, TN-Fα, ADIPOQ **D**, IL-8, and MCP1 **E** expression in hMADS adipocytes co-cultured with macrophages. **F** IL-1β concentration (pg/ml) measured by ELISA in cell co-culture supernatant. **G** Representative immunoblot and quantification of pJNK expression in macrophages co-cultured with hMADS adipocytes. Data are expressed as mean ± SEM or mean ± SD. *p≤0.05; **p≤0.01; ***p≤0.001; ^#^*p*≤0.05; ^##^*p*≤0.01; ^##^*p*≤0.001. *vs M0; #vs hMADS

To disentangle the dynamic interplay between macrophages and adipocytes in vitro, we set up a co-culture system of THP-1-derived M0, M1, M2, and HgSM macrophages and differentiated hMADS adipocytes. These latter after 12 to 15 days of differentiation show an abundant multilocular lipid content that can be easily visualized by oil red O staining (Suppl Fig. 3A) and express the typical markers of mature white adipocytes [27]. THP-1 monocytic cells were seeded onto transwell inserts, differentiated into M0, M1, M2, and HgSM macrophages, then co-cultured with differentiated hMADS adipocytes for 48 h (Suppl Fig. 3B). Real-time PCR analysis of HgSM cells co-cultured with hMADS adipocytes showed a significantly higher expression of pro-inflammatory cytokines TNFα, IL-8, and IL-6, together with a significantly reduced expression of IL-10 mRNA, in comparison to M0 cells (Fig. 5A). As expected, M1 macrophages showed the typical increased expression of IL-6 mRNA together with low IL-10 mRNA level (Fig. 5A). IL-1β protein expression was significantly increased in HgSMs co-cultured with hMADS (Fig. 5B). Since the most relevant proinflammatory cytokines are produced under the control of the NF-kB proinflammatory pathway, we analyzed IKB-α protein, inhibitor of NF-kB signal [29]. A significantly reduced expression of IKB-α protein was observed in HgSMs compared to M0 cells (Fig. 5B), as well as in hMADS adipocytes co-cultured with HgSMs (Fig. 5C). An increased mRNA expression of the p65 NF-kB subunit was observed in hMADS co-cultured with all the macrophage phenotypes (Suppl.Fig. 3C). Overall, these results suggest that HgSMs can worsen the pro-inflammatory activity induced by NF-kB signaling activation in adipocytes, a condition that could be observed in vivo when macrophages infiltrate adipose tissue. In accordance with this hypothesis, hMADS adipocytes co-cultured with HgSM macrophages showed a significant hyper-expression of the pro-inflammatory cytokines IL-6, IL-1β, TNF-α, IL-8, and MCP1 together with a significant downregulation of adiponectin (ADIPOQ) mRNA compared not only to hMADS adipocytes cultured alone (control) but also to hMADS co-cultured with M0 cells (Fig. 5D and Fig. 5E). In accordance, hMADS adipocytes co-cultured with HgSMs secreted a significative higher concentration of IL-1β compared to hMADS adipocytes co-cultured with M0 (Fig. 5F).

A reduced SIRT1 mRNA expression and protein levels was observed in hMADS co-cultured with all the macrophage phenotypes compared to the control adipocytes (Suppl Fig. 3C and Suppl Fig. 3D).

Finally, since C-Jun NH (2)-terminal kinase (JNK) signaling pathway is involved in the modulation of the inflammatory process and in the metabolic response to obesity, including insulin resistance [30], we analyzed THP-1-derived macrophages for JNK phosphorylation by Western blotting. Our analysis showed that, on one hand, only HgSM cells co-cultured with hMADS adipocytes exhibited increased JNK phosphorylation compared to M0 cells (Fig. 5G). On the other hand, hMADS co-cultured with M0, M1, M2, and HgSM macrophages exhibited increased JNK phosphorylation compared to the control hMADS (Suppl Fig. 3E). Collectively, these data suggest that the co-culture of HgSM macrophages and hMADS adipocytes (1) moves senescent HgSMs toward a pro-inflammatory phenotype, and (2) moves hMADS adipocytes toward a proinflammatory phenotype, characterized by reduced expression of adipokines and increased release of cytokines.

### Effect of HgSM macrophages on insulin signaling in hMADS adipocytes

There is a close and bidirectional link between obesity, chronic inflammation, and insulin resistance. Adipose tissue inflammation, involving increased infiltration of M1 pro-inflammatory macrophages, adipocyte death and abnormal cytokine and adipokine secretion, is a major actor of obesity-induced chronic systemic inflammation and insulin resistance [31]. On the other hand, adipose tissue is an important target of insulin and an impairment of insulin signaling in subcutaneous and visceral fat importantly worsen body insulin sensitivity. Thus, we assessed the effects of HgSM macrophages on hMADS adipocyte insulin signaling by measuring the IRS1 protein levels and the phosphorylation of the 473serine kinase AKT (pAKT), a central player in the insulin signaling pathway [32].

**Figure 6:**
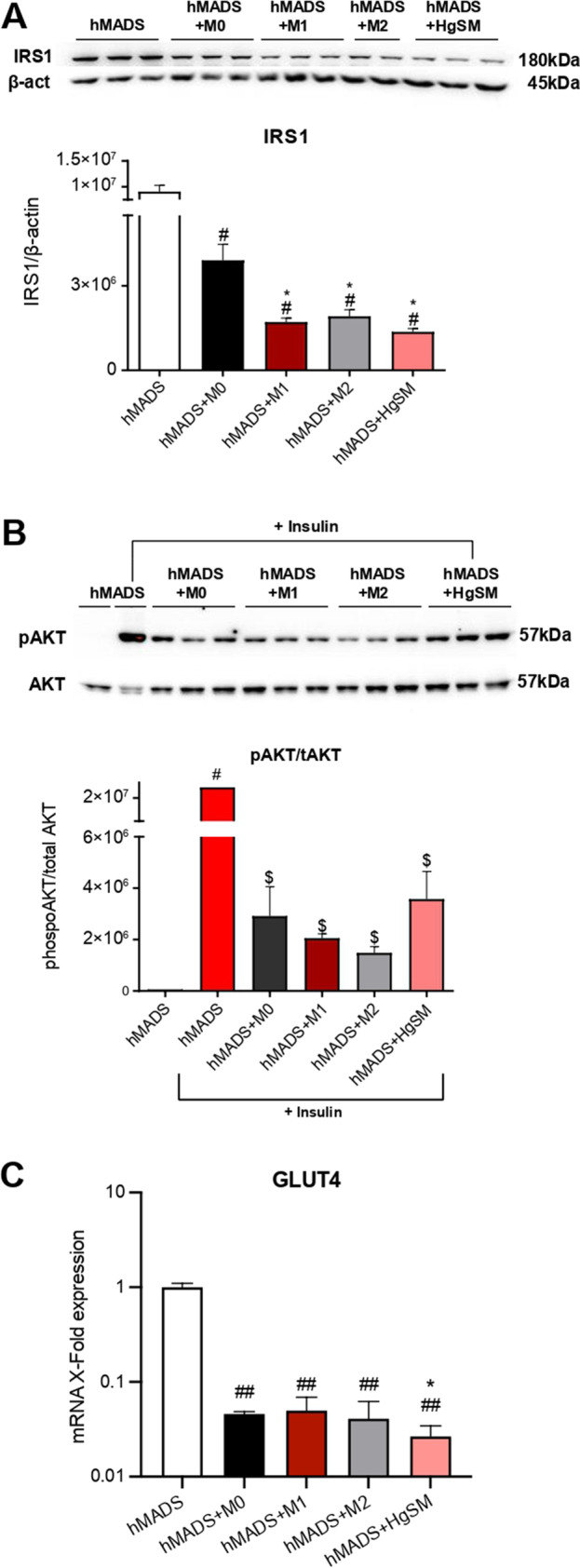
Effect of macrophages on insulin signaling in hMADS adipocytes. Representative immunoblot and quantification of IRS1 (**A**) and pAKT (**B**) in hMADS adipocytes co-cultured with macrophages. **C** qRT-PCR analysis of GLUT4 expression in hMADS adipocytes co-cultured with macrophages. Data are expressed as mean ± SEM, *p≤0.05; **p≤0.01; ***p≤0.001; ^#^*p*≤0.05; ^##^*p*≤0.01; ^###^*p*≤0.001, ^$^*p*≤0.05; ^$$^*p*≤0.01; ^$$$^*p*≤0.001. *vs M0; #vs hMADS; $vs hMADS+Insulin

Western blot analysis demonstrated that hMADS exhibited a significantly reduced IRS1 expression in each experimental condition that was evident in HgSM-hMADS co-cultures (Fig. 6A).

With the purpose of analyzing the pAKT insulin-dependent signaling, hMADS adipocytes were co-cultured with M0, M1, M2, and HgSM macrophages for 48 h, and then incubated with 100 nM insulin for 20 min before cell lysis, followed by the analysis of pAKT expression. Importantly, HgSMs induced a variable but significant reduction of pAKT level in response to 100 nM insulin. The pAKT insulin-dependent signaling was also downregulated in M0-, M1-, and M2-treated hMADS adipocytes in comparison with insulin-treated hMADS adipocytes (Fig. 6B).

Finally, GLUT4 mRNA levels were examined. As shown in Fig. 6C, a notable reduction in GLUT4 levels was observed in hMADS adipocytes co-cultured with M0, M1, M2, and HgSM macrophages. Of note, HgSM-co-cultured adipocytes displayed a lower GLUT4 expression compared to M0 co-cultured adipocytes (Fig. 6C). Taken together, these results suggest that macrophages may functionally regulate insulin signaling and that HgSMs may play a critical role in decreasing insulin signal transduction in human adipocytes.

### MiRNAs expression in adipose tissues samples, in HgSMs and hMADS

To explore if the observed senescent phenotype of the adipose tissue of obese subjects is paralleled also by the rearrangement of miRNAs deregulated by senescence, we measured the expression levels of miR-146a and miR-21 in the same adipose tissue samples of obese subjects, and in cellular models**.**

**Figure 7:**
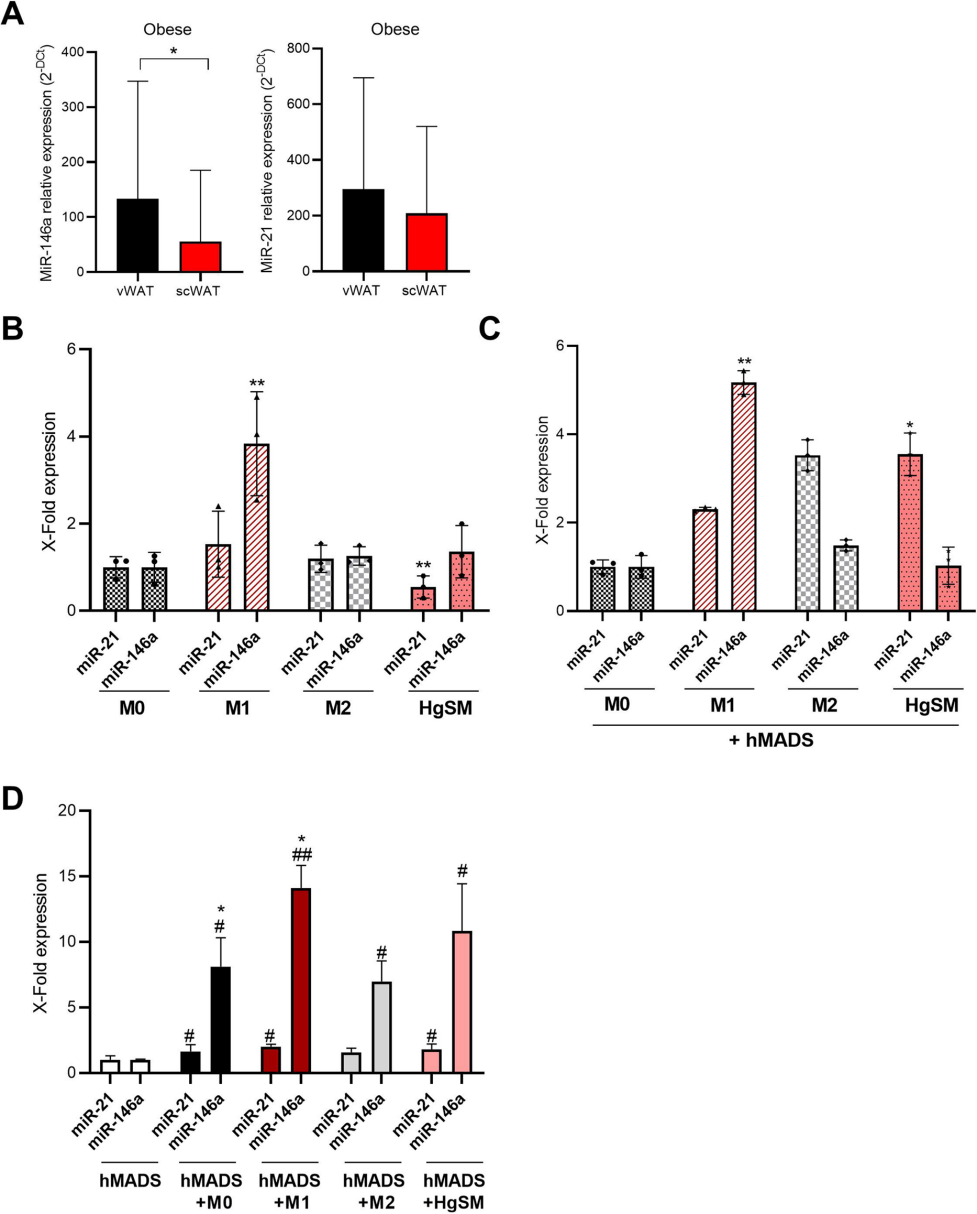
Analysis of miRNA expression in adipose tissue, THP-1-induced macrophages, and hMADS adipocytes in co-cultured condition. **A** miRNA expression analysis by RT-PCR of miR-146a and miR-21 in vWAT and scWAT. **B** miRNA expression analysis by qRT-PCR of miR-21 and miR-146a in M0, M1, M2, and HgSM macrophages. **C** miRNA expression analysis by qRT-PCR of miR-21 and miR-146a in M0, M1, M2, and HgSM macrophages co-cultured with hMADS adypocytes. **D** miRNA expression analysis by qRT-PCR of miR-21 and miR-146a in hMADS adipocytes co-cultured with M0, M1, M2, and HgSM macrophages. Data are expressed as mean ± SD, *p≤0.05; **p≤0.01; ***p≤0.001; ^#^*p*≤0.05; ^##^*p*≤0.01; ^##^*p*≤0.001. *vs M0; #vs hMADS

In adipose tissue samples, we observed a significant increased level of miR-146a in vWAT compared with scWAT (Fig. 7A). Further, miRNA expression analysis in macrophages differentiated in M1, M2, and HgSM confirmed a significant hyperexpression of miR-146a in M1 compared to M0, underlining the pro-inflammatory condition of M1 (Fig. 7B). In HgSMs, miR-21 levels were significantly reduced compared to M0 (Fig. 7B), whereas in HgSMs co-cultured with hMADS adipocytes the expression levels of miR-21 were significantly increased (Fig. 7C).

Finally, adipocytes co-cultured with all macrophage phenotypes expressed higher level of miR-146a, whereas only HgSMs induce a low but significant increase of miR-21 compared to control adipocytes (Fig. 7D).

Overall, these results suggest that miR-146a is hyperexpressed in vWAT samples, confirming its role as anti-inflammatory miRNA that attempts to restrain a proinflammatory condition.

In the cellular model, the co-culture of HgSMs with hMADS adipocytes is associated with an increased expression level of miR-21 in HgSMs as well as a higher level of miR-21 and miR-146a in hMADS.

These data confirmed the pro-inflammatory crosstalk between macrophages and adipocytes.

## Discussion

Obesity is a powerful risk factor for T2DM and a major source of chronic and systemic inflammation [33]. Increasing evidence strongly points at the involvement of SCs in fueling inflammation and unbalancing insulin sensitivity [21]. In this investigation, a significant higher number of SA-β-Gal + cells was observed in vWAT compared to scWAT of obese patients. Moreover, SA-β-Gal + cells number in vWAT but not in scWAT was positively correlated with BMI, HOMA index, and serum insulin level. Owing to the role of inflammation in insulin resistance [34] and due to the expected role of senescence-related secretome in systemic inflammation [35], it can be speculated that the accumulation of SCs within visceral adipose tissue depots might trigger insulin resistance and promote T2DM. Of note, the evaluation of SA-β-Gal + cells in a small group of normal-weight individuals has revealed that the SA-β-Gal + cells were significantly lower in vWAT of normal weight controls compared to obese patients.

Such data suggest that the accrual observed in obese subjects might be of pathological relevance.

We also observed that the majority of SCs (about 70% of the total) in vWAT and scWAT were positive for the pan-macrophage marker CD68. Pre-adipocytes and T cells may concur to the 30% of SA-β-Gal + cells negative for CD68 in obese individuals, as suggested by previous studies [25, 36]. Senescent status of SA-β-Gal + cells was also confirmed by p16^INK4a^ nuclear staining. Indeed, SCs have been analyzed in human abdominal and femoral subcutaneous fat biopsies from healthy subjects, showing an increased number of SCs in the subcutaneous abdominal adipose tissue, and an association with inflammatory signals [37]. Another study found an increased abundance of senescence markers in mesenchymal stromal cells harvested from subcutaneous fat tissue of obese subjects when compared to lean controls [38]. In addition, one report suggested that SCs present in thigh fat are associated with physical function [39]. Of note, SCs present in adipose tissue are emerging as druggable targets, given the ability of selected drugs to promote their clearance both in vivo and in ex vivo explants [40].

As adipose tissue composition is heterogeneous, the individual role of senescent cells within this tissue is debated [9, 41]. Preadipocytes can senesce, either via proliferative exhaustion during aging or by increased oxidative stress promoted by obesity [22, 42]. Interestingly, also chronic hyperinsulinemia has been recently suggested to drive adipocyte senescence through cell cycle re-entry [43]. Endothelial cells are present in the WAT vascular system and have been also suggested to acquire a senescent phenotype in obese subjects [44]. The vWAT of obese and old mice and humans is also enriched in immune cells with characteristics of cellular senescence, as T cells, the pro-inflammatory ABCs, and macrophages [45]. Interestingly, also peripheral immune cells from patients with either obesity or T2DM are characterized by an accelerated senescent phenotype, extending the observations obtained in the adipose tissue at the systemic level [46, 47].

To our knowledge, our study, for the first time, conveys the presence of senescent macrophages within human vWAT and scWAT samples from the same subject. Notably, macrophages expressing senescence markers have been previously found to physiologically occur in the adipose tissue of aged mice [26, 48, 49]. However, it has been hypothesized that senescent macrophages can fuel inflammaging [50]. Since we recently have demonstrated a direct link between hyperglycemia and the senescent-associated secretory phenotype (SASP) in endothelial cells and macrophages [10], in this study, we aimed to characterize the senescence status acquired by macrophages in high-glucose milieu, and how it can be modulated by adipocyte crosstalk. After a week of high-glucose-medium exposure, a senescent phenotype in THP-1-derived macrophages and a polarization toward a mixed M1/M2-like phenotype were observed. We called this peculiar macrophage phenotype as high-glucose-induced senescent macrophage (HgSM) and we speculated that this may represent a phenomenon that occurs in vivo when macrophages are exposed to hyperglycemia. HgSMs are characterized by the simultaneous increased release of inflammatory and anti-inflammatory mediators, such as TNF-α and IL-10, indicative of a condition that has been termed as immunoparalysis, as it occurs in patients affected by sepsis and septic shock [51, 52].

Of note, a mixed pro- and anti-inflammatory phenotype was previously observed in both macrophages with senescent features [10], but also in senescent endothelial cells [53, 54]. Worth mentioning, albeit the SASP is usually accompanied by a set of shared core proteins, the resulting secretory phenotype of SCs is highly dependent of the pro-senescence stimulus but also of the cell type involved [55].

A shift of circulating monocytes in aged people toward non-canonical pro-inflammatory phenotypes has been previously described [56, 57]. Moreover, the phenotype of aged macrophages resembles that of type 2 anti-inflammatory macrophages, partially reprogrammed toward a pro-inflammatory phenotype [58].

Because systemic inflammation with aging depends on a complex balance between inflammaging and anti-inflammaging mechanisms, the complex metabolic reshape occurring during aging (macroph-aging) may turn to be crucial to explain the onset of (adipose) tissue inflammation and to describe its systemic effects [50, 59, 60].

During the progression of obesity, an accumulation of macrophages and other immune cells occurs in adipose tissue [61, 62], and this global reshape of tissue resident immune cells may contribute to the adipose tissue inflammatory derangement that occurs with aging, particularly in obese subjects [63]. Indeed, the interaction of HgSMs with adipocytes could affect their features and polarization. The co-culture of THP-1-derived macrophages with hMADS adipocytes revealed a shift toward a proinflammatory status in presence of HgSMs. To disentangle the molecular mechanisms involved in such proinflammatory shift, we analyzed some molecules belonging to NF-kB pathway, the main pro-inflammatory signaling in human cells [64]. Notably, the NF-kB signaling pathway is also involved in sensitivity/resistance to insulin [65]. NF-kB activity is mediated by the activation of the IKK complex, which in turn leads to IKBα degradation with the consequent release of the NF-kB dimers that translocate in the nucleus modulating target gene expression [29]. The modulation of the IKB-α protein confirmed the presence of pro-inflammatory metabolic switch in HgSMs when co-cultured with hMADS.

Interestingly, also hMADS adipocytes showed an important upregulation of several pro-inflammatory factors, such as IL-6, IL-1β, TNF-α, IL-8, and MCP1 when co-cultured with HgSM macrophages, corroborating the notion that macrophage secretome can foster bystander, low-grade inflammation. Consistently, in HgSMs-treated hMADS adipocytes, the insulin signaling pathway was substantially inhibited. In insulin-treated hMADS adipocytes co-cultured with HgSMs, we observed a significant reduction of pAKT levels, thus point at an activity of that HgSMs-derived factors to inhibit pAKT insulin-dependent signaling [66]. This effect of HgSMs on hMADS adipocytes was accompanied by a marked reduction in IRS1 and GLUT4 expression. TNF-α is known to impair insulin signaling in adipocytes by decreasing Akt levels, and levels of molecules that mediate the effects of insulin, including IRS1 and GLUT4 [67–69]. In addition, IL-6 secreted by macrophages also impairs insulin signaling in adipocytes and multiple animal models [70]. Furthermore, IL-1β released by macrophages mediates macrophage-induced impairment of insulin signaling pathway in human primary adipocytes [71]. Hence, a complex pro-inflammatory secretome from HgSMs may concur in setting off insulin resistance in hMADS. Our data showed that HgSM macrophages co-cultured with hMADS adipocytes exhibited an increased JNK phosphorylation. c-Jun NH (2)-terminal kinase (JNK) signaling pathway contributes to inflammation and to play a key role in the metabolic reshape associated with obesity [30]. In particular, JNK-deficient macrophages of mice fed with a high-fat diet remained insulin-sensitive and JNK inactivation in myeloid cells led to reducing macrophage accumulation in the adipose tissue and the expression of M1 cytokines [30]. The data above support the notion that JNK signaling pathway may be involved in the induction of insulin resistance in human adipocytes exposed to HgSMs.

SIRT1 has been shown to protect against obesity; indeed, the ablation in animal model of SIRT1 in adipocyte or macrophages exacerbates obesity-induced metabolic dysregulation [72, 73]. Moreover, it is known that SIRT1 in adipocytes modulates expression of several adipokines including adiponectin and MCP1 [72]. In line with these findings, our data showed that HgSM macrophages promoted in hMADS adipocytes a reduction of SIRT1 accompanied by an increased expression of MCP1 and reduced expression of adiponectin.

Finally, we analyzed the expression levels of some inflammation-related microRNAs, named inflammamiRs [13], in HgSMs and in vWAT compared to scWAT samples of obese subjects. We observed a significantly reduction of miR-21 expression in HgSMs compared to M1 and M2 differentiated THP-1 cells. Interestingly, the co-culture with hMADS was associated with an increased expression of miR-21 in HgSMs. These results are in line with recent evidence suggesting that the overexpression of miR-21 significantly upregulates the pro-inflammatory cytokines, pushing the cells toward a pro-inflammatory phenotype, with partial involvement of NF-kB signal pathway [74].

In tissue samples of obese patients, we observed a significant increased level of miR-146a in vWAT compared to scWAT, suggesting that miR-146a overexpression may be a mechanism to restrain inflammation [75]. This mechanism seems not efficient in HgSMs and hMADS adipocytes, suggesting a derailment toward an inflammatory status. Speculatively, other (immune) cell types different from macrophages and adipocytes could be involved in miR-146a hyper-expression in vWAT.

## Conclusion

Obesity is a major trigger of chronic inflammation, promoting the development of diabetes. However, no druggable target halting this pathological process has emerged so far [76]. Among others, SCs have been suggested as potential mediators of obesity-induced inflammation. Here we demonstrate for the first time that a sizeable amount of SCs in the adipose tissue of obese human subjects are macrophages (SA-β-Gal and CD68 + cells) and that the number of SCs resident in the vWAT correlate with IR. These results, coupled with the observed ability of in vitro reproduced senescent macrophages to spread inflammation and promote IR in hMADS, corroborate a framework where SCs, and in particular senescent macrophages, might contribute to adipose tissue inflammation and diabetes development. Given that SCs are fast becoming druggable targets [77] and considering that SC removal has already been shown to improve glucose tolerance and enhance insulin sensitivity in obese mice [11], the results presented here encourage the design of further studies [40] testing the effect of SCs removal, or other safe strategies reducing the deleterious effect of SCs, on diabetes-related endpoints in obese subjects.

## Supplementary Information

Below is the link to the electronic supplementary material.

ESM 1 (DOCX 906 KB)

## Data Availability

All data generated or analyzed during this study are included in this published article.
