# Supplementary material for: Senescent macrophages in the human adipose tissue as a source of inflammaging
Source: GeroScience. 2022 Mar 5;44(4):1941–60. doi: 10.1007/s11357-022-00536-0 (PMC9616990; doi:10.1007/s11357-022-00536-0)

**Supplementary Information**

Supplementary Figure 1

**A** Spearman correlation between number of SA-β-Gal + cells and BMI (i) (r=0.020; p=0.938), HOMA-index (ii) (r=0.192, p=0.460) and Fasting insulin (iii) (r=0.211; p=0.417) in scWAT. Data are expressed as mean ± SD, *p≤0.05; **p≤0.01; ***p≤0.001. (JPG 181 KB)


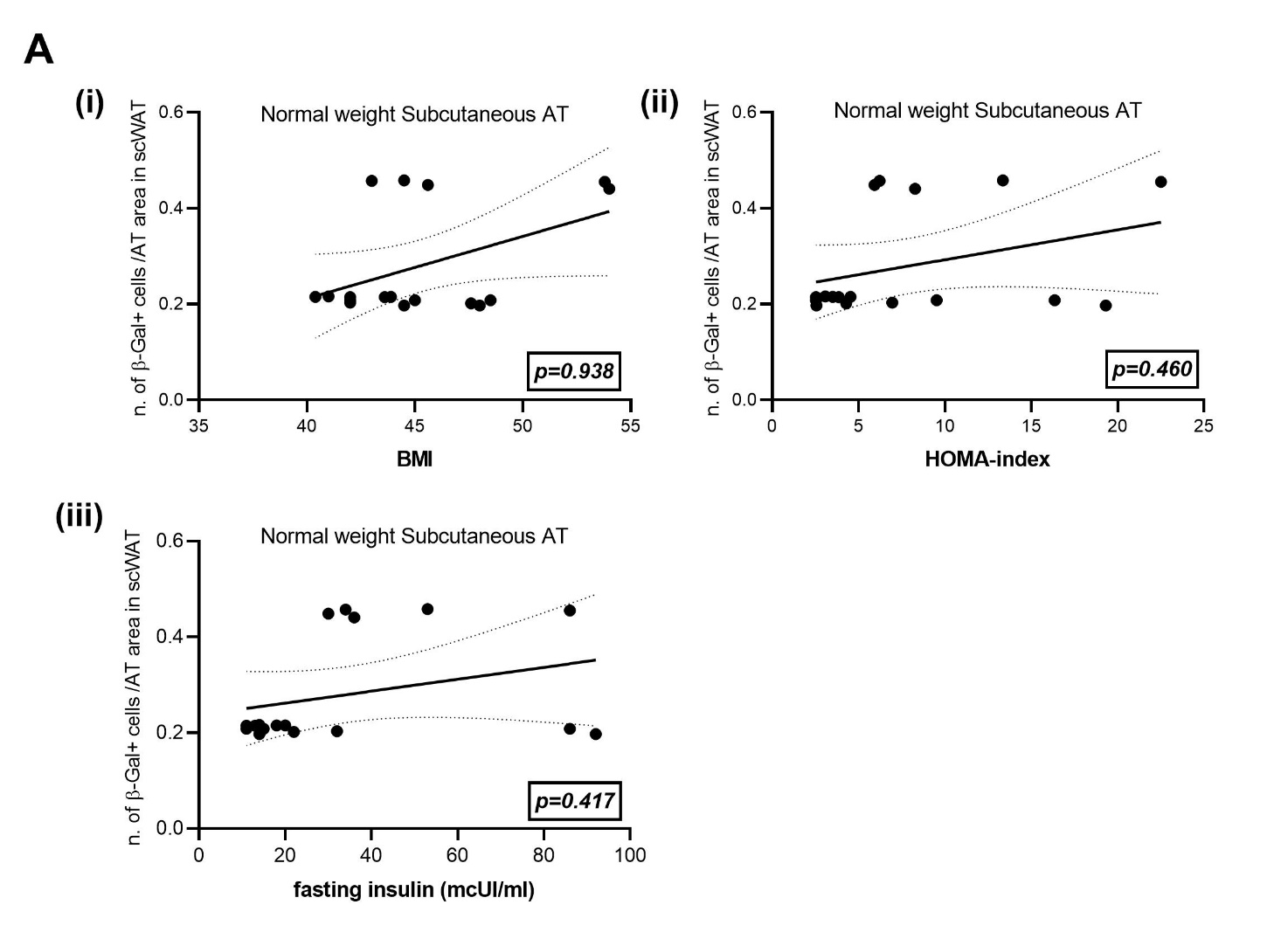


Supplementary Figure 2

Senescent macrophages in obese adipose tissue. Representative microscopy picture of a crown-like structure (CLS) in vWAT of obese subjects showing double positive (SA-β-Gal+/p16 +) cells (arrows). Bar =10 μm; Bar inset =5 μm.


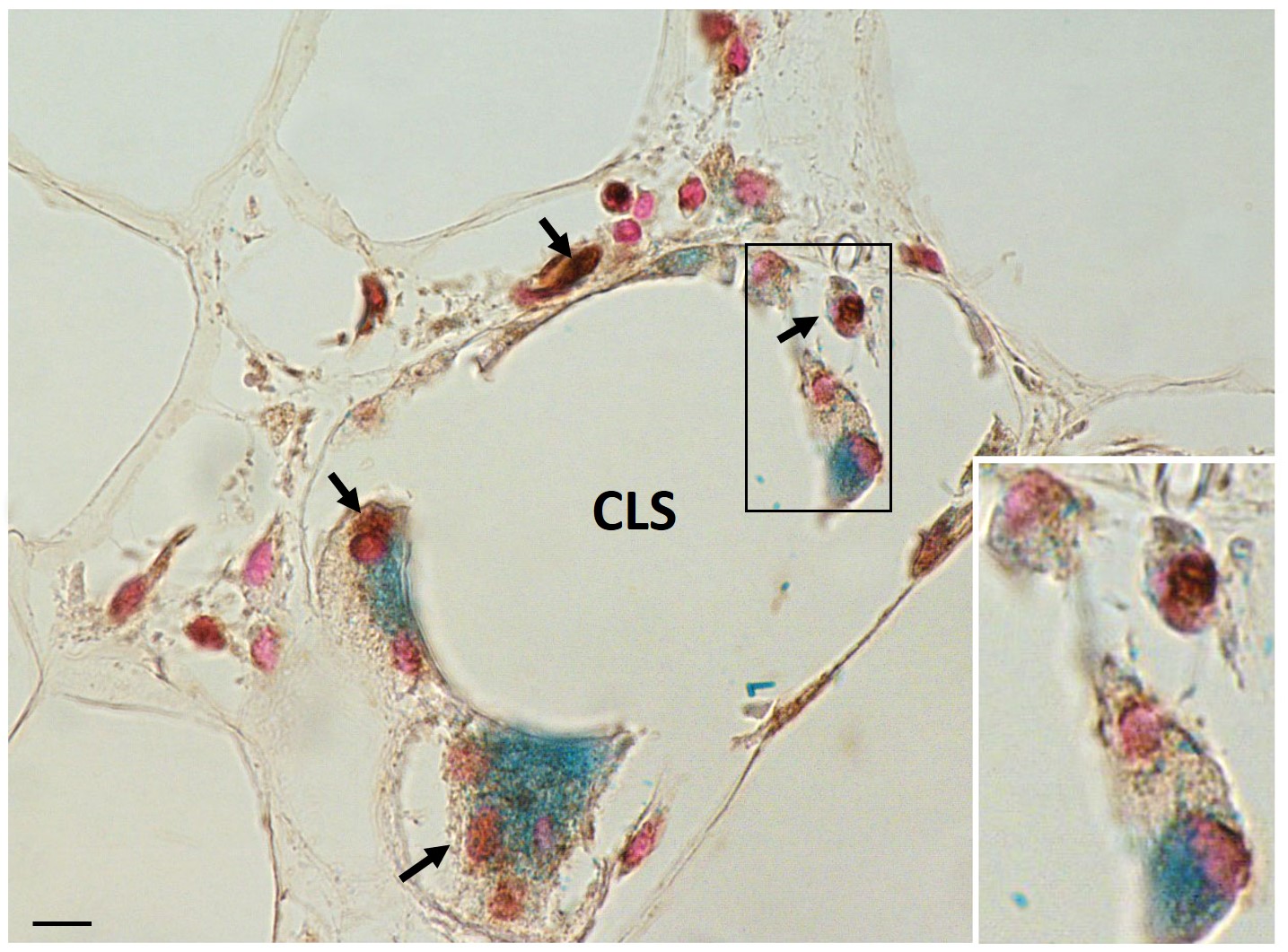


Supplementary Figure 3

**A** Representative pictures from Oil Red O-stained confluent hMADS cells (day 0, upper panel) and full differentiated adipocytes (day 13, bottom panel). **B** Transwell system for the co-culture experiment (THP-1 cells in the insert above; hMADS cells in the lower chamber). **C** qRT-PCR analysis of SIRT1 and NF-kB expression in hMADS adipocytes co-cultured with macrophages. Representative immunoblot and quantification of SIRT1 **D** and pJNK **E** in hMADS adipocytes co-cultured with macrophages. Data are expressed as mean ± SEM, *p≤0.05; **p≤0.01; ***p≤0.001; #p≤0.05; ##p≤0.01; ##p≤0.001. *vs M0; #vs hMADS.


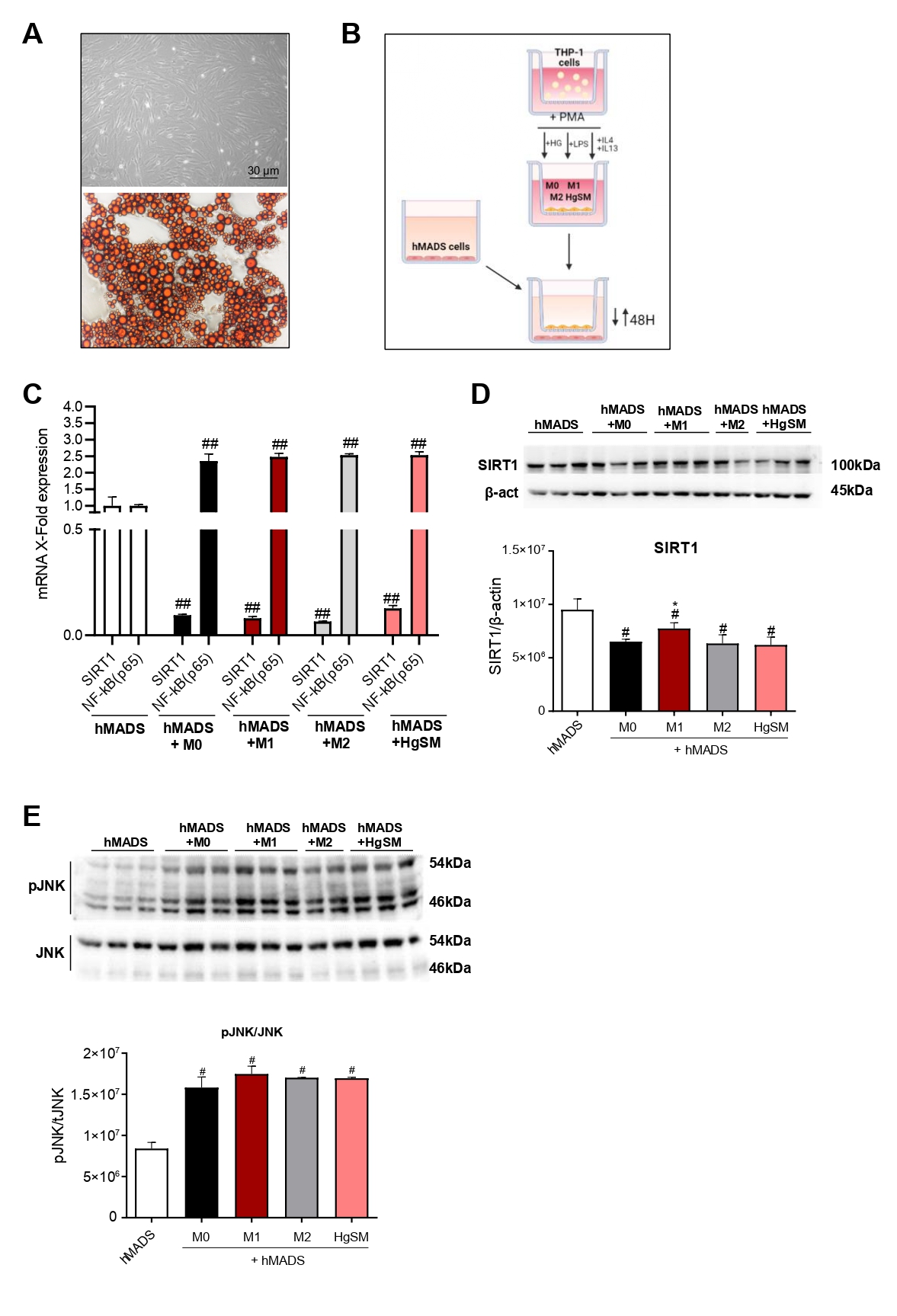

Supplement: Supplementary file 1 — (DOCX 906 KB) [file 11357_2022_536_MOESM1_ESM.docx]
